# Association Between the Creatinine-to-Body Weight Ratio and All-Cause Mortality in a Rural Japanese Community

**DOI:** 10.7759/cureus.96746

**Published:** 2025-11-13

**Authors:** Ryuichi Kawamoto, Asuka Kikuchi, Daisuke Ninomiya, Masanori Abe, Teru Kumagi

**Affiliations:** 1 Department of Community Medicine, Ehime University Graduate School of Medicine, Tōon, JPN; 2 Postgraduate Medical Education Center, Ehime University Hospital, Tōon, JPN

**Keywords:** all-cause mortality, annual health checkups, cohort study, community-dwelling persons, creatinine/body weight ratio, japanese, risk factors, rural communities

## Abstract

Background

Evidence on the association between the creatinine-to-body weight (Cr/BW) ratio and the risk of all-cause mortality remains limited. In this prospective, population-based cohort study, we examined the association between baseline Cr/BW ratio and survival over 10- and 22-year follow-up periods.

Methods

Participants included 1553 men (aged 63 ± 14 years) and 1964 women (aged 65 ± 16 years) who participated in the Nomura cohort study conducted in 2002 (first cohort) and 2014 (second cohort) and who continued throughout the follow-up periods (median, 6316 days; interquartile ranges, 3828-8121 days). Participants were categorized into four groups based on the baseline Cr/BW ratio (×100) standard deviation ranges: 0.53-0.96 (Cr/BW ratio-1), 0.97-1.25 (Cr/BW ratio-2), 1.26-1.62 (Cr/BW ratio-3), and 1.63-7.34 (Cr/BW ratio-4). Adjusted relative risk estimates for all-cause mortality were obtained using data from the Basic Resident Register. Cox proportional hazards regression analysis was performed, with the time variable defined as the period between age at recruitment and age at death or censoring. The model included covariates such as age, sex, body height, smoking and alcohol habits, and histories of cardiovascular disease, hypertension, dyslipidemia, diabetes, chronic kidney disease, and hyperuricemia.

Results

For the 3517 participants, 1196 deaths (34.0%) were documented, including 604 men (38.9% of men) and 592 women (30.1% of women). Analysis of the Cr/BW ratio as a continuous variable revealed a significant positive association with all-cause mortality risk (hazard ratio (HR), 1.73; 95% confidence interval (CI), 1.28-2.35). In multivariate Cox models, the adjusted HRs (95% CI) for mortality relative to the lowest Cr/BW category (ratio-1) were 1.33 (1.07-1.66), 1.36 (1.08-1.70), and 1.59 (1.20-2.11) for ratio-2, ratio-3, and ratio-4, respectively (*p* for trend = 0.015). Furthermore, considering both sensitivity and specificity, the optimal cutoff value of the Cr/BW ratio for predicting all-cause mortality was determined to be 1.23 (sensitivity, 63.0%; specificity, 56.0%).

Conclusion

The study demonstrated that the Cr/BW ratio serves as a significant predictor of subsequent mortality risk. Strategies aimed at keeping the Cr/BW ratio within a specific range could contribute to reducing overall mortality.

## Introduction

Sarcopenia, defined by a decline in muscle mass, strength, and function, has increasingly attracted attention from clinical researchers due to its link with numerous chronic conditions [1], frailty, disability, and poor quality of life [2]. Recent studies have reported a significant association between total muscle mass and strength and the risk of all-cause mortality [3,4].

Muscle mass is typically evaluated using advanced methodologies such as dual-energy X-ray absorptiometry [5] and bioelectrical impedance analysis [6]. In some studies, trunk muscle mass is estimated from cross-sectional areas on magnetic resonance imaging (MRI) or computed tomography (CT) [7,8]. However, these approaches require specialized equipment, are costly, and, in the case of CT, involve radiation exposure. Consequently, they are not practical for large-scale or routine clinical assessments. Therefore, there is growing interest in developing simple, accessible, and reliable surrogate markers of muscle mass and body composition. Ideally, a blood-based marker combined with basic anthropometric data (height, weight, and sex) could provide a practical estimate of lean body mass, including skeletal muscle mass and fat mass. Serum creatinine (Cr), which is produced at a relatively constant rate and primarily determined by total muscle mass [9], has been proposed as such a marker. In individuals with normal kidney function, Cr serves as a cost-effective and readily available proxy for evaluating skeletal muscle mass [9].

The Cr/BW ratio has been introduced as a simple indicator reflecting the skeletal muscle mass index (SMI) adjusted for body weight (BW). Previous research has shown that this ratio more accurately predicts mortality than BMI, fat mass, or fat mass index [10]. Moreover, the creatinine/(cystatin C × BW) ratio has been associated with weight-adjusted SMI, further supporting its role as a relative muscle mass marker [11]. Recent studies have demonstrated that the Cr/BW ratio predicts type 2 diabetes mellitus [12], non-alcoholic fatty liver disease [13,14], and all-cause mortality [15]. Interestingly, the correlation between creatinine levels and body weight appears to be even stronger, and negative, among non-obese individuals [16]. Nevertheless, the relationship between the Cr/BW ratio and all-cause mortality has not been fully elucidated. While prior studies have focused on disease-specific outcomes or limited subpopulations, evidence linking Cr/BW to overall mortality risk in the general adult population remains scarce. Clarifying this association is critical for establishing Cr/BW as a clinically meaningful and easily obtainable biomarker of health risk.

Accordingly, this study pursued two main objectives: (1) to analyze the association between the Cr/BW ratio and multiple risk factors and (2) to evaluate the relationship between Cr/BW and all-cause mortality.

## Materials and methods

Study design and population

This prospective, population-based cohort study is part of the Nomura Study [17,18], which was initiated in 2002 for the first cohort and in 2014 for the second. Participants were recruited from rural communities in Ehime Prefecture, Japan, specifically individuals who routinely participated in annual community-based health checkups conducted at the Nomura Health and Welfare Center. Reference values employed in the analysis were primarily based on established findings from previous studies. The first cohort consisted of 3164 participants, and the second cohort included 1832 individuals, all aged between 22 and 95 years. The study was conducted in strict accordance with relevant ethical standards and regulatory requirements. The research protocol received approval from the Institutional Review Board (IRB) of Ehime University Hospital (IRB no. 1903018), and all participants provided written informed consent.

**Figure 1:**
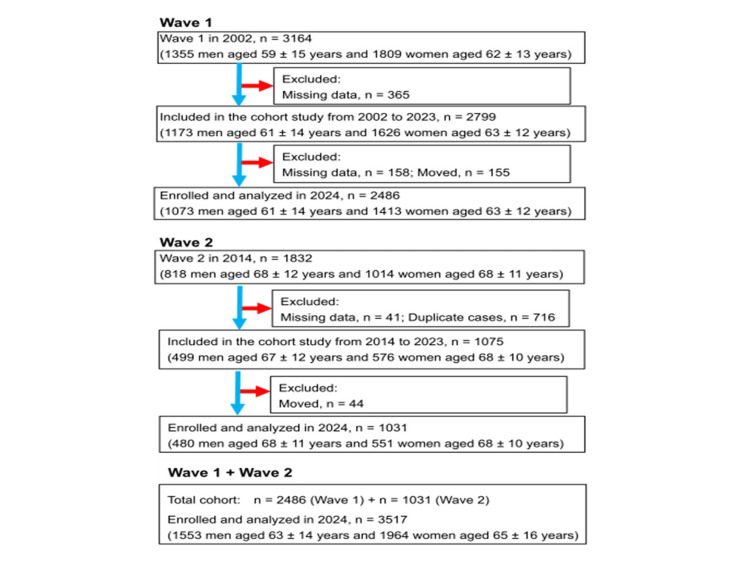
Flowchart of participants The first cohort consisted of 3164 participants, and the second cohort included 1832 individuals, all aged between 22 and 95 years. Follow-up assessments were carried out 22 years after the enrollment of the first cohort and 10 years after that of the second cohort. Data from both cohorts were combined for analysis, yielding a total sample size of 3517 individuals.

Participants’ medical histories, current health status, and medication use were assessed through structured, questionnaire-based interviews (Appendix A). Figure 1 shows a flowchart illustrating the inclusion and exclusion criteria used for participant selection. Missing data were addressed using complete-case analysis. Follow-up assessments were conducted 22 years after enrollment for the first cohort and 10 years after enrollment for the second cohort. Residency status was verified using Japan’s Basic Resident Register. Data from both cohorts were combined for analysis, resulting in a total sample size of 3517 participants (Appendix B).

Evaluation of risk factors

All anthropometric measurements were conducted in accordance with World Health Organization (WHO) guidelines [19]. BW was measured to the nearest 0.1 kg using a digital scale (HBF-214; Omron, Tokyo, Japan), with participants wearing only underwear or light clothing. Body height (BH) was measured to the nearest 1 cm using a stadiometer (DSN-90; Muratec-KDS, Kyoto, Japan) while participants stood barefoot. Body mass index (BMI) was determined by dividing BW (kg) by the square of body height (m²). Exposure to cigarette smoking was assessed as the product of smoking duration (in years) and the average daily cigarette consumption. Participants were stratified into four groups according to smoking history: non-smokers, former smokers, light smokers (<20 pack-years), and heavy smokers (≥20 pack-years) [20]. Alcohol consumption was assessed using the Japanese liquor unit, equivalent to 22.9 grams of ethanol per unit. Participants were grouped into four categories: non-drinkers, occasional drinkers (<1 unit/day), light daily drinkers (1-2 units/day), and heavy daily drinkers (2-3 units/day), with no participants exceeding a daily intake of 3 units [18]. Blood pressure, including systolic and diastolic measurements, was obtained using an automated device (BP-103i; Colin, Aichi, Japan). All measurements were conducted in the seated position following a minimum five-minute rest, with the right upper arm fitted with an appropriately sized cuff. For analysis, the mean of two consecutive blood pressure measurements was used. After an overnight fast, blood samples were collected for biochemical assessment of triglycerides (TG), high-density lipoprotein cholesterol (HDL-C), low-density lipoprotein cholesterol (LDL-C), serum uric acid (SUA), creatinine (Cr) (enzymatic method), and blood glucose (BG). The Cr/BW ratio was calculated by dividing Cr by BW, while the Cr-to-BH (Cr/BH) ratio was determined by dividing Cr by BH. Similarly, the Cr to BMI (Cr/BMI) ratio was calculated by dividing Cr by BMI. The estimated glomerular filtration rate (eGFR) was calculated using the Chronic Kidney Disease Epidemiology Collaboration (CKD-EPI) formula, adjusted with a Japanese coefficient. Estimated values were calculated using sex-specific and creatinine-dependent equations. For men, the formula was 141 × (Cr/0.9) ^− 0.411 ^× 0.993 ^age^ × 0.813, for Cr ≤ 0.9 mg/dL, and 141 × (Cr/0.9) ^− 1.209 ^× 0.993 ^age^ × 0.813, for Cr > 0.9 mg/dL. For women, the corresponding equations were 144 × (Cr/0.7) ^− 0.329^ × 0.993 ^age^ × 0.813, for Cr ≤ 0.7 mg/dL, and 144 × (Cr/0.7) ^− 1.209^ × 0.993 ^age^ × 0.813, for Cr > 0.7 mg/dL [21].

Hypertension was defined as SBP ≥ 140 mmHg, DBP ≥ 90 mmHg, or being on current antihypertensive therapy [22]. Hypertriglyceridemia was defined as TG ≥ 150 mg/dL, hypo-HDL cholesterolemia as HDL-C < 40 mg/dL, and high-LDL cholesterolemia as LDL-C ≥ 140 mg/dL or use of lipid-lowering medication [23]. Diabetes was defined as BG ≥ 126 mg/dL or treatment with antidiabetic agents [24]. Hyperuricemia was defined as SUA ≥ 7.0 mg/dL or administration of SUA-lowering medications [25]. Chronic kidney disease (CKD) was defined as eGFR ≤ 60 mL/min/1.73 m² [26]. Cardiovascular disease (CVD) was defined to include ischemic heart disease, ischemic stroke, and peripheral vascular disease.

Statistical analysis

Statistical analyses were carried out using IBM SPSS Statistics, version 28.0 (IBM Corp., Armonk, USA) or JMP Trial 18.1.1 (JMP Statistical Discovery LLC, USA). Continuous variables that followed a normal distribution were expressed as means ± standard deviations (SDs), whereas non-normally distributed variables, such as TG and BG levels, were presented as medians with interquartile ranges (IQRs). To ensure normality, variables that deviated from a normal distribution underwent logarithmic transformation, and these transformed values were employed in the subsequent analyses.

To identify predictors of all-cause mortality, the area under the receiver operating characteristic (ROC) curve was calculated for each variable. The ROC curve is a graphical tool that plots sensitivity (true positive rate) against 1 − specificity (false positive rate) for each marker. An ideal ROC curve would trace a path from (0,0) to (0,1) and then from (0,1) to (1,1). In practice, the ROC curve typically falls between these two extremes, with the area under the curve (AUC) serving as a summary measure of diagnostic accuracy across the full range of test values. A diagonal line, representing an AUC near 0.5, indicates a test with no discriminatory power. To identify the optimal cutoff points for all-cause mortality, the Youden index was computed as sensitivity + specificity − 1, with the value yielding the maximum Youden index taken as the optimal cutoff.

Subjects were divided into four groups based on the SD of the baseline Cr/BW ratio (×100) (Cr/BW ratio-1, 0.53-0.96; Cr/BW ratio-2, 0.97-1.25; Cr/BW ratio-3, 1.26-1.62; Cr/BW ratio-4, 1.63-7.34). Group differences in continuous variables were assessed using Student’s t-test or analysis of variance (ANOVA), and categorical variables were compared using the χ² test. Cox proportional hazards regression was used to estimate hazard ratios (HRs) and 95% confidence intervals (CIs), with age as the time scale. The entry time was defined as age at baseline (in days), and the exit time as age at death or at the end of follow-up, whichever occurred first. The proportional hazards assumption was visually assessed through log-minus-log survival plots, with no major violations detected. Four sequential models were specified: Model 1, unadjusted; Model 2, adjusted for age and sex; Model 3, additionally adjusted for BMI, smoking and alcohol habits, and history of CVD; and Model 4, further adjusted for hypertension, hypertriglyceridemia, hypo-HDL cholesterolemia, high-LDL cholesterolemia, diabetes, CKD, and hyperuricemia. Multicollinearity among covariates was assessed using the variance inflation factor. Restricted cubic spline (RCS) analysis was conducted to evaluate the linearity and dose-response relationship between the Cr/BW ratio (×100) and all-cause mortality, adjusting for Model 4 covariates. Knots were placed at the 5th, 35th, 65th, and 95th percentiles of the Cr/BW distribution.

Subgroup analyses stratified by sex, age (<65 vs. ≥65 years), BMI (<25 vs. ≥25 kg/m²), CKD status, and time to death were performed to assess the robustness of the association. Interaction analyses were conducted to evaluate effect modification, adjusting for all confounders except the interaction variable. Statistical significance was defined as *p* < 0.05.

## Results

The cohort comprised 3517 participants, with a mean age of 64 ± 12 years, of whom 44.2% were male. The median follow-up period was 6316 days (IQR, 3828-8121 days). During the follow-up, 1196 participants (34.0%) died, including 604 men (38.9% of male participants) and 592 women (30.1% of female participants). The number of events relative to the number of covariates included in the multivariate Cox model yielded a high events-per-variable ratio (= 80), indicating that model estimates were statistically stable.

AUC values (95% CIs) for anthropometric indices used to predict all-cause mortality

**Figure 2:**
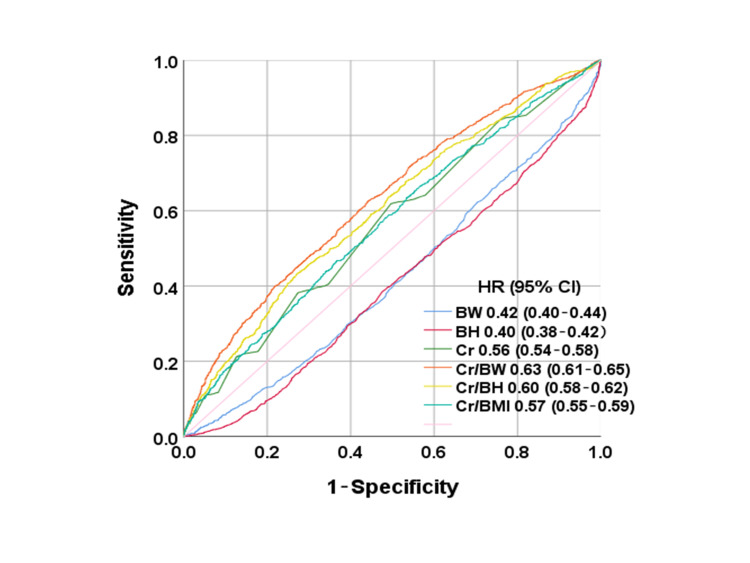
AUC values (95% CI) for anthropometric indices used to predict all-cause mortality The area under the curves (AUCs) for individual anthropometric indices (body weight (BW), body height (BH), body mass index (BMI), creatinine (Cr), Cr to BW (Cr/BW) ratio, Cr to BH (Cr/BH) ratio, and Cr to BMI (Cr/BMI) ratio) in predicting all-cause mortality were calculated.

Figure 2 presents the AUC for individual anthropometric indices (BW, BH, BMI, Cr, Cr/BW ratio, Cr/BH ratio, and Cr/BMI ratio) used to predict all-cause mortality. Of these anthropometric indices, the Cr/BW ratio yielded the greatest AUC (0.63; 95% CI, 0.61-0.65).

Baseline characteristics of participants stratified by the baseline Cr/BW ratio

The baseline characteristics of the participants, stratified by the Cr/BW ratio, are summarized in Table 1. Among the Cr/BW ratio groups, a higher Cr/BW ratio was associated with a higher proportion of males, older age, smoking habits, a history of CVD, and a higher prevalence of hypertension, CKD, and hyperuricemia. Conversely, BH, BW, BMI, hypertriglyceridemia, and hyper-LDL cholesterolemia were lower in individuals with higher Cr/BW ratios.

**Table 1:** Baseline characteristics of participants stratified by baseline Cr/BW ratios Cr, creatinine; BW, body weight; HDL, high-density lipoprotein; LDL, low-density lipoprotein; eGFR, estimated glomerular filtration rate; SUA, serum uric acid. All data are presented as means ± standard deviations unless otherwise indicated. Triglycerides and blood glucose, which exhibited skewed distributions, are reported as median (interquartile range) and were log-transformed for analyses. ^*^p-values were calculated using ANOVA for continuous variables and χ² tests for categorical variables.

	Total	Cr/BW ratio-1	Cr/BW ratio-2	Cr/BW ratio-3	Cr/BW ratio-4	*p*-value*
	0.53–7.34	0.53–0.96	0.97–1.25	1.26–1.62	1.63–7.34	
Baseline characteristics	n = 3517	n = 524	n = 1340	n = 1244	n = 409	
Gender (male), n (%)	1553 (44.2)	124 (23.7)	544 (40.6)	656 (52.7)	229 (56.0)	<0.001
Age (years)	64 ± 12	58 ± 12	62 ± 12	66 ± 12	73 ± 9	<0.001
Body height (m)	1.55 ± 0.09	1.56 ± 0.09	1.56 ± 0.10	1.55 ± 0.09	1.52 ± 0.09	<0.001
Body weight (kg)	56.2 ±10.5	63.3 ± 11.3	57.8 ± 10.0	53.7 ± 8.9	49.4 ± 8.6	<0.001
Body mass index (kg/m^2^)	23.3 3.2	26.0 ± 3.5	23.7 ± 2.8	22.3 ± 2.6	21.3 ± 2.7	<0.001
Smoking habits						
Non-smokers (%)	71.6	80.9	73.6	67.0	67.5	<0.001
Former smokers (%)	18.9	15.5	18.6	20.2	20.0	
Light smokers (%)	4.6	1.5	3.8	6.4	5.9	
Heavy smokers (%)	4.9	2.1	4.0	6.4	6.6	
Drinking habits						
Non-drinkers (%)	51.5	53.1	49.6	50.6	58.7	0.001
Occasional drinkers (%)	25.9	29.0	27.6	24.0	22.5	
Light daily drinkers (%)	13.6	11.6	12.8	15.6	12.5	
Heavy daily drinkers (%)	9.0	6.3	9.9	9.9	6.4	
History of cardiovascular disease (%)	279 (7.9)	32 (6.1)	80 (6.0)	106 (8.5)	61 (14.9)	<0.001
Hypertension, n (%)	1995 (56.7)	292 (55.7)	715 (53.4)	709 (57.0)	279 (68.2)	<0.001
Systolic blood pressure (mmHg)	137 ± 21	138 ± 21	137 ± 21	137 ± 22	141 ± 21	<0.001
Diastolic blood pressure (mmHg)	80 ± 11	81 ± 12	81 ± 11	80 ± 11	79 ± 11	0.033
Use of antihypertensive medication, n (%)	1097 (31.2)	140 (26.7)	366 (27.3)	411 (33.0)	180 (44.0)	<0.001
Hypertriglyceridemia, n (%)	638 (18.1)	111 (21.2)	277 (20.7)	191 (15.4)	59 (14.4)	<0.001
Triglycerides (mg/dL)	93 (70–131)	97 (71–141)	95 (71–136)	90 (68–126)	89 (68–123)	<0.001
Hypo-HDL cholesterolemia, n (%)	151 (4.3)	22 (4.2)	74 (5.5)	38 (3.1)	17 (4.2)	0.022
HDL cholesterol (mg/dL)	63 ± 16	61 ± 15	62 ± 16	64 ± 16	63 ± 16	0.003
Hyper-LDL cholesterolemia, n (%)	1109 (31.5)	173 (33.0)	436 (32.5)	384 (30.9)	116 (28.4)	0.345
LDL cholesterol (mg/dL)	118 ± 31	122 ± 31	119 ± 32	117 ± 30	115 ± 31	<0.001
Use of lipid-lowering medication, n (%)	341 (9.7)	46 (8.8)	120 (9.0)	131 (10.5)	44 (10.8)	0.414
Diabetes, n (%)	446 (12.7)	79 (15.1)	143 (10.7)	157 (12.6)	67 (16.4)	0.005
Blood glucose (mg/dL)	100 (91–114)	100 (91–117)	99 (90–114)	101 (91–116)	103 (92–118)	<0.001
Use of anti-diabetic medication, n (%)	312 (8.9)	59 (11.3)	102 (7.6)	104 (8.4)	47 (11.5)	0.017
Chronic kidney disease, n (%)	355 (10.1)	0	10 (0.7)	119 (9.6)	226 (55.3)	<0.001
eGFR (mL/min/1.73 m^2^)	77.9 ± 16.3	96.3 ± 15.2	82.6 ± 11.6	72.3 ± 10.5	56.4 ± 12.5	<0.001
Hyperuricemia	485 (13.8)	28 (5.3)	151 (11.3)	198 (15.9)	108 (26.4)	<0.001
SUA (mg/dL)	5.2 ± 1.4	4.8 ± 1.2	5.0 ± 1.4	5.3 ± 1.4	5.8 ± 1.5	<0.001
Use of SUA-lowering medication, n (%)	151 (4.3)	8 (1.5)	50 (3.7)	57 (4.6)	36 (8.8)	<0.001

Threshold effect analysis of baseline Cr/BW ratio in relation to all-cause mortality

**Figure 3:**
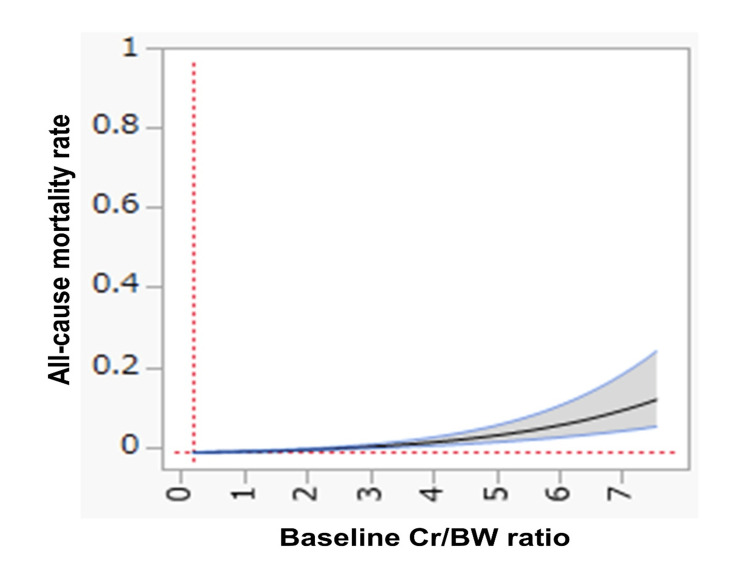
Threshold effect analysis of the baseline Cr/BW ratio in relation to all-cause mortality Cr, creatinine; BW, body weight. Using restricted cubic spline (RCS) regression, and after adjusting for potential confounders, we found a positive association between the Cr/BW ratio and all-cause mortality.

Threshold effect analysis was performed to assess the dose-response relationship between the baseline Cr/BW ratio and all-cause mortality. Using RCS regression adjusted for potential confounders, a positive association was observed between the Cr/BW ratio and mortality (Figure 3). Furthermore, considering both sensitivity and specificity (Figure 1), the optimal cutoff value of the Cr/BW ratio for predicting all-cause mortality was determined to be 1.23 (sensitivity, 63.0%; specificity, 56.0%).

Kaplan-Meier survival curves for the overall survival of participants stratified by baseline Cr/BW ratio

**Figure 4:**
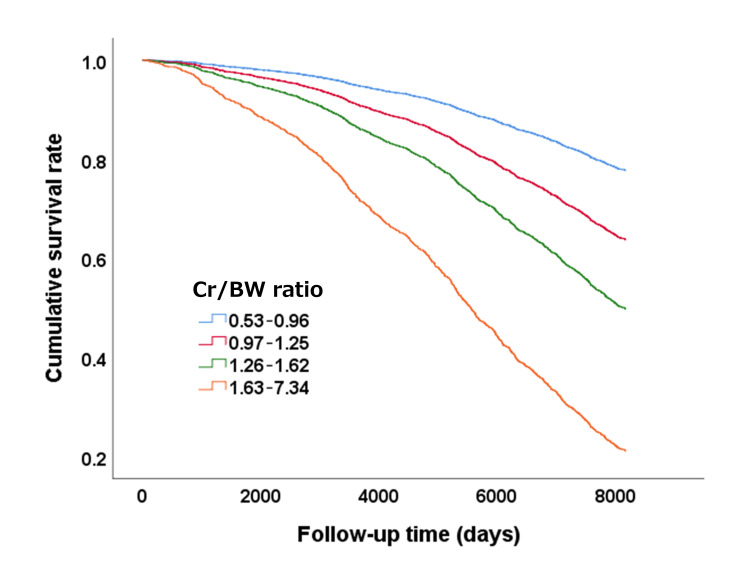
Kaplan–Meier survival curves for the overall survival of participants stratified by the baseline Cr/BW ratio Cr, creatinine; BW, body weight A significantly lower survival rate was observed for a larger Cr/BW ratio, and this trend was maintained across the entire duration of the observation period (log-rank test, *p *< 0.001).

Kaplan-Meier survival estimates were plotted to compare survival across Cr/BW ratio categories (Figure 4). A significantly lower survival rate was observed in individuals with higher Cr/BW ratios, and this trend persisted over the entire follow-up period (log-rank test, *p* < 0.001).

Hazard ratios and 95% confidence intervals of the baseline Cr/BW ratio (categorical data) for all-cause mortality

Multivariate analysis, adjusted for confounders, indicated that the Cr/BW ratio was significantly linked to elevated all-cause mortality risk (Table 2). A higher Cr/BW ratio (×100) (continuous data) was associated with an elevated HR for all-cause mortality (HR, 1.73; 95% CI, 1.28-2.35). The HRs for all-cause mortality, compared with the Cr/BW ratio-1 (0.53-0.96), were 1.33 (95% CI, 1.07-1.66) for the Cr/BW ratio-2 (0.97-1.25), 1.36 (95% CI, 1.08-1.70) for the Cr/BW ratio-3 (1.26-1.62), and 1.59 (95% CI, 1.20-2.11) for the Cr/BW ratio-4 (1.63-7.34) (*p* for trend = 0.015).

**Table 2:** HRs and 95% CIs of baseline Cr/BW ratio for all-cause mortality HR, hazard ratio; CI, confidence interval; HDL, high-density lipoprotein; LDL, low-density lipoprotein; Cr, creatinine; BW, body weight Four sequential models were constructed: Model 1, unadjusted; Model 2, adjusted for age and sex; Model 3, additionally adjusted for body height, alcohol consumption, and history of cardiovascular disease; and Model 4, further adjusted for hypertension, hypertriglyceridemia, hypo-HDL cholesterolemia, high-LDL cholesterolemia, diabetes, chronic kidney disease, and hyperuricemia, incorporating all covariates from Model 3. ^*^Continuous data.

	Total	Cr/BW ratio-1	Cr/BW ratio-2	Cr/BW ratio-3	Cr/BW ratio-4	*p* for trend
	0.53–7.34	0.53–0.96	0.97–1.25	1.26–1.62	1.63–7.34	
Characteristics	n = 3517	n = 524	n = 1340	n = 1244	n = 409	
Prevalence of death, n (%)	1196 (34.0)	100 (19.1)	394 (29.4)	467 (37.5)	235 (57.5)	<0.001
Person-days of follow-up, median (interquartile range) days	6316 (3828–8121)	8099 (4431–8155)	7713 (3856–8153)	5209 (3801–8107)	3856 (3525–6100)	<0.001
Model 1, HR (95% CI)	6.29 (5.22–7.53)*	Reference	1.79 (1.43–2.22)	2.77 (2.23–3.44)	6.16 (4.86–7.80)	<0.001
Model 2, HR (95% CI)	1.79 (1.41–2.29)*	Reference	1.31 (1.05–1.63)	1.39 (1.11–1.74)	1.67 (1.30–2.15)	0.001
Model 3, HR (95% CI)	1.71 (1.33-2.19)*	Reference	1.32 (1.05–1.64)	1.36 (1.09–1.71)	1.62 (1.25–2.09)	0.003
Model 4, HR (95% CI)	1.73 (1.28–2.35)*	Reference	1.33 (1.07–1.66)	1.36 (1.08–1.70)	1.59 (1.20–2.11)	0.015

Hazard ratios and 95% confidence intervals of the baseline Cr/BW ratio (continuous data) for all-cause mortality by sub-analysis

Table 3 presents HRs and 95% CIs for the baseline Cr/BW ratio in relation to all-cause mortality, stratified by sex, age, BMI, CVD history, CKD status, and time to death. The association between a higher Cr/BW ratio and increased all-cause mortality was observed consistently across sex, age (≥65 years), BMI (<25.0 kg/m^2^), CKD status (No), and time to death (≥1095 days). A history of CVD was identified as a significant effect modifier (*p* = 0.002, for interaction). Consistent with previous analyses, participants with a time to death ≥1095 days and a higher Cr/BW ratio exhibited an elevated risk of all-cause mortality.

**Table 3:** HRs and 95% CIs of baseline Cr/BW ratio (continuous data) for all-cause mortality by sub-analysis HR, hazard ratio; CI, confidence interval; Cr, creatinine; BW, body weight The model was adjusted for all covariates in Model 4 of Table 2.

Characteristics (N = 3517)	HR (95% CI)	*p*-value	*p* for interaction
Gender			
Men (n = 1553)	1.62 (1.03–2.55)	0.037	0.149
Women (n = 1964)	1.85 (1.23–2.80)	0.003	
Age			
<65 years (n = 1509)	1.26 (0.63–2.53)	0.519	0.913
≥65 years (n = 2008)	1.84 (1.32–2.58)	<0.001	
Body mass index			
<25.0 kg/m^2^ (n = 2569)	1.87 (1.28–2.73)	0.001	0.981
≥25.0 kg/m^2^ (n = 948)	1.72 (0.86–3.42)	0.125	
History of cardiovascular disease			
No (n = 3238)	1.86 (1.35–2.57)	<0.001	0.002
Yes (n = 279)	0.97 (0.41–2.31)	0.949	
Chronic kidney disease			
No (n = 3162)	1.55 (1.10–2.20)	0.013	0.532
Yes (n = 355)	2.12 (1.15–3.89)	0.016	
Time to death			
<1095 days (n = 75)	3.09 (0.88–10.8)	0.078	--
≥1095 days (n = 3442)	1.58 (1.16–2.17)	0.004	

## Discussion

This cohort study demonstrates that the baseline Cr/BW ratio is an independent and significant predictor of all-cause mortality among community-dwelling adults. In multivariate analyses, participants in Cr/BW ratio groups 2 through 4 (0.97-7.34) showed significantly higher hazard ratios compared with those in group 1 (0.53-0.96), after adjustment for potential confounders. Furthermore, considering both sensitivity and specificity, the optimal cutoff value of the Cr/BW ratio for predicting all-cause mortality was determined to be 1.23. The association was confirmed through multifactorial analyses. To mitigate potential reverse causality, individuals who died within three years of follow-up were excluded, with results remaining largely consistent. Subgroup analyses further substantiated the robustness of these findings. To date, few studies have investigated the association between baseline Cr/BW ratio and all-cause mortality in Japanese community-dwelling populations.

Few studies have explored the association between the Cr/BW ratio and all-cause mortality [15]. An analysis utilizing prospective data from the National Health and Nutrition Examination Survey (NHANES) demonstrated a U-shaped association. The threshold effect analysis identified 0.96 as the cutoff point for the Cr/BW ratio (×100). Below this value, higher ratios were associated with lower all-cause mortality (HR, 0.63; 95% CI, 0.41-0.97), whereas above 0.96, higher ratios were linked to increased mortality risk (HR, 1.67; 95% CI, 1.41-1.97) [15]. These conflicting results may be attributed to the aging process, which involves a decline in muscle mass and a shift in body fat distribution. There have been several reports on the relationship between Cr/BW and outcomes other than mortality [12-14,27,28]. Recent studies have identified a reduced Cr/BW ratio as a newly recognized biomarker that is significantly linked to the development of non-alcoholic fatty liver disease in the general population [13]. Additionally, new evidence suggests a negative and nonlinear correlation between the Cr/BW ratio and diabetes risk among the general Japanese population [12] and Chinese populations [27,28]. Reports suggesting a negative association have been based on studies in populations with a mean age of under 50 years. Similarly, in line with the findings of He et al. [15], we also observed no association in the group under 65 years of age.

Creatinine, a metabolic waste product derived from muscle breakdown, serves as an indicator of kidney function but is also influenced by muscle mass. Since the Cr/BW ratio reflects skeletal muscle quantity [29], which is a major component of lean body mass [30], its impact may vary across different age groups [31]. In younger individuals, an elevated Cr/BW ratio indicates greater skeletal muscle mass [32] and a lower risk of mortality. In contrast, aging is associated with a decline in muscle mass and shifts in body fat distribution [33]. Older adults are at a higher risk of developing chronic diseases that can lead to weight loss and increased mortality risk [34]. Relative skeletal muscle mass offers a more accurate representation of the interaction between fat, bone, and lean body mass, potentially providing a clearer understanding of the relationship between obesity and mortality [15]. A higher Cr/BW ratio may reflect lower body fat levels and diminished energy reserves. Additionally, serum Cr serves as a marker of renal function and tends to increase as GFR declines. As a result, the Cr/BW ratio in older adults is positively correlated with mortality. Our study found that a higher Cr/BW ratio was associated with an increased HR for all-cause mortality, independent of renal function.

This study benefited from an extended follow-up period, which allowed for more precise hazard ratio estimations. Furthermore, the sensitivity analysis that excluded mortality data from the initial three years produced results consistent with the primary findings, reinforcing their robustness. Nevertheless, several considerations should be taken into account when interpreting these outcomes. First, the study population primarily consisted of middle-aged and older adults from a rural Japanese community (mean age, 64 ± 12 years), which may limit the generalizability of the findings to other populations. Second, the analysis included only individuals recorded as deceased in the basic resident register, thereby excluding those who relocated during the follow-up period. Third, factors such as concomitant medications, preexisting health conditions (e.g., chronic diseases, inflammation, and nutritional status), and lifestyle variables (e.g., dietary habits, physical activity, and weight changes) at baseline and throughout the study were not comprehensively assessed and may have influenced the observed associations. Although we attempted to account for potential confounders using baseline physical examination data, residual confounding due to unmeasured factors cannot be ruled out. Fourth, the combination of two cohorts recruited 12 years apart introduces potential cohort heterogeneity that is not accounted for. Finally, despite its strengths, this study is subject to inherent limitations and potential biases related to external validity. In particular, selection bias due to participant loss from relocation and the focus on a rural Japanese population may restrict the applicability of the findings to other demographic or geographic settings.

## Conclusions

The baseline Cr/BW ratio is an independent predictor of all-cause mortality among community-dwelling adults. This association remained robust after adjustment for confounding factors, exclusion of early deaths, and subgroup analyses. These findings highlight the potential utility of the Cr/BW ratio as a simple and practical clinical marker for long-term mortality risk.

## Appendices

Appendix A

**Table 4:** The specific health checkup questionnaire

受診券整理番号 Checkup ticket number	
お名前 Name	
生年月日 Date of birth	年 月 日 Y M D
記入日（受診日 Today's date	年 月 日 Y M D

質問項目 Questionnaire		選択肢 0ptions
1	血圧を下げる薬を服用している。 Do you take any medication to reduce blood pressure?	①はい ②いいえ ① Yes ② No
2	インスリン注射又は血糖を下げる薬を服用している。 Do you take insulin injection or medication to reduce blood sugar?	①はい ②いいえ ① Yes ② No
3	コレステロールを下げる薬を服用している。（中性脂肪含む） Do you take medication to reduce your cholesterol level?	①はい ②いいえ ① Yes ② No
4	医師から、脳卒中（脳出血、脳梗塞等）にかかっているといわれたり、治療を受けたことがありますか。 Have you ever been told by the doctor you have a stroke? (cerebral hemorrhage, brain infarction, etc.) and received treatment?	①はい ②いいえ ① Yes ② No
5	医師から、心臓病（狭心症、心筋梗塞等）にかかっているといわれたり、治療を受けたことがありますか。 Have you ever been told by the doctor you have a heart disease? (angina pectoris, myocardial infarction, etc.) and received treatment?	①はい ②いいえ ① Yes ② No
6	医師から、慢性の腎不全にかかっているといわれたり、治療（人工透析）を受けたことがありますか。 Have you ever been diagnosed as having a chronic kidney failure or received treatment (dialysis therapy)?	①はい ②いいえ ① Yes ② No
7	医師から、貧血といわれたことがある。 Have you ever been diagnosed you were anemic?	①はい ②いいえ ① Yes ② No
8	現在たばこを習慣的に吸っている。 （※ 「現在、習慣的に喫煙している者」とは、条件１と条件２を両方満たす者である。条件１：最近１か月間吸っている 条件２：生涯で６か月間以上吸っている、又は合計１００本以上吸っている） Currently a habitual smoker. (Meets both Condition 1 and Condition 2), Condition 1: Smoking for the last 1 month. Condition 2: Smoking for more than 6 months in your lifetime or more than 100 cigarettes in total.	①はい（条件１と条件２を両方満たす） Yes（Meets both Condition 1 and Condition 2） ②以前は吸っていたが、最近１か月間は吸っていない（条件２のみ満たす） ②I used to smoke, but I haven’t smoked for the last 1 month (only Condition 2 is satisfied). ③いいえ（① ②以外） ③ No
9	お酒（日本酒、焼酎、ビール、洋酒など）を飲む頻度はどのくらいですか 飲酒日の１日当たりの飲酒量日本酒１合（アルコール度数 15 度・180mL）の目安：ビール（同 5 度・500mL）、焼酎（同 25 度・約 110mL）、ワイン（同 14 度・約 180mL）、ウイスキー（同 43 度・60mL）、缶チューハイ（同 5 度・約 500mL、同 7 度・約 350mL) How often do you drink alcohol (sake, shochu, beer, western liquor, etc.)? Approximate amount of alcohol consumed per day on drinking days 1 gou of sake (15% alcohol by volume, 180 mL): beer (5 degrees, 500 mL), shochu (25 degrees, approx. 110 mL), wine (14 degrees, approx. 180 mL), whiskey (43 degrees, 60 mL), canned chuhai (5 degrees, approx. 500 mL, 7 degrees, approx. 350 mL)	① 飲まない 時々 毎日 ① non-drinkers occasional drinkers daily drinkers １合未満（<1 unit/day） 1～2合未満 （1–2 units/day）2～3合未満（2–3 units/day）3合以上（more than 3 units/day）

Appendix B

**Table 5:** Baseline characteristics of participants stratified by the first cohort and second cohort HDL, high-density lipoprotein; LDL, low-density lipoprotein; eGFR, estimated glomerular filtration rate; SUA, serum uric acid. All data are presented as means ± standard deviations unless otherwise indicated. Triglycerides and blood glucose, which exhibited skewed distributions, are reported as median (interquartile range) and were log-transformed for analyses. ^*^*p*-values were calculated using ANOVA for continuous variables and χ² tests for categorical variables.

	1st cohort	2nd cohort	*p*-value*
Baseline characteristics (N = 3517)	n = 2486	n = 1031	
Gender (male), n (%)	1073 (43.2)	480 (46.6)	0.068
Age (years)	62 ± 13	68 ± 11	<0.001
Body height (m)	1.55 ± 0.09	1.56 ± 0.10	0.017
Body weight (kg)	56.4 ±10.4	55.8 ± 10.7	0.176
Body mass index (kg/m^2^)	23.4 ± 3.2	22.9 ± 3.2	<0.001
Smoking habits			
Non-smokers (%)	1801 (72.4)	718 (69.6)	0.070
Former smokers (%)	465 (18.7)	198 (19.2)	
Light smokers (%)	101 (4.1)	62 (6.0)	
Heavy smokers (%)	119 (4.8)	53 (5.1)	
Drinking habits			
Non-drinkers (%)	1094 (44.0)	718 (69.6)	<0.001
Occasional drinkers (%)	713 (28.7)	199 (19.3)	
Light daily drinkers (%)	439 (17.7)	39 (3.8)	
Heavy daily drinkers (%)	240 (9.7)	75 (7.3)	
History of cardiovascular disease (%)	215 (8.6)	64 (6.2)	0.016
Hypertension, n (%)	1354 (54.5)	641 (62.2)	<0.001
Systolic blood pressure (mmHg)	138 ± 22	135 ± 18	<0.001
Diastolic blood pressure (mmHg)	81 ± 12	78 ± 10	
Use of antihypertensive medication, n (%)	671 (27.0)	426 (41.3)	<0.001
Hypertriglyceridemia, n (%)	478 (19.2)	160 (15.5)	0.009
Triglycerides (mg/dL)	95 (71–134)	87 (67–125)	<0.001
Hypo-HDL cholesterolemia, n (%)	151 (6.1)	0	<0.001
HDL cholesterol (mg/dL)	61 ± 15	66 ± 17	<0.001
Hyper-LDL cholesterolemia, n (%)	670 (27.0)	439 (42.6)	<0.001
LDL cholesterol (mg/dL)	117 ± 32	120 ± 30	0.009
Use of lipid-lowering medication, n (%)	139 (5.6)	202 (19.6)	<0.001
Diabetes, n (%)	235 (9.5)	211 (20.5)	<0.001
Blood glucose (mg/dL)	95 (88–103)	117 (108–126)	<0.001
Use of anti-diabetic medication, n (%)	235 (9.5)	77 (7.5)	0.068
Chronic kidney disease, n (%)	243 (9.1)	112 (10.9)	0.326
eGFR (mL/min/1.73 m^2^)	80.0 ± 17.5	73.0 ± 11.7	<0.001
Hyperuricemia	336 (13.5)	149 (14.5)	0.485
SUA (mg/dL)	5.1 ± 1.4	5.3 ± 1.4	<0.001
Use of SUA-lowering medication, n (%)	107 (4.3)	44 (4.3)	1.000
Creatinine/body weight (×100)	1.20 (1.03-1.40)	1.30 (1.12-1.51)	<0.001
